# *N*′-(4-Fluoro­benzyl­idene)-2-(4-fluoro­phen­yl)acetohydrazide

**DOI:** 10.1107/S1600536811039845

**Published:** 2011-10-05

**Authors:** Hoong-Kun Fun, Madhukar Hemamalini, V. Sumangala, G. K. Nagaraja, Boja Poojary

**Affiliations:** a X-ray Crystallography Unit, School of Physics, Universiti Sains Malaysia, 11800 USM, Penang, Malaysia; b Department of Chemistry, Mangalore University, Mangalagangothri 574 199, Mangalore, Karnataka, India

## Abstract

In the title compound, C_15_H_12_F_2_N_2_O, the dihedral angle between the two benzene rings is 48.73 (8)°. The hydrazine group is twisted slightly, with a C—N—N—C torsion angle of 172.48 (12)°. In the crystal, mol­ecules are connected by strong N—H⋯O and weak C—H⋯O hydrogen bonds, forming supra­molecular chains along the *c* axis. The structure is consolidated by π–π [centroid–centroid separation = 3.6579 (10) Å] and C—H⋯π inter­actions.

## Related literature

For further details of aroyl­hydro­zones, see: Li & Qu (2011 ▶); Zhang (2011 ▶); Fan *et al.* (2008 ▶). Ajani *et al.* (2010 ▶); Avaji *et al.* (2009 ▶); Rasras *et al.* (2010 ▶).
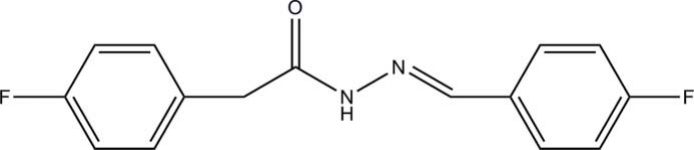


## Experimental

### 

#### Crystal data

C_15_H_12_F_2_N_2_O
*M* *_r_* = 274.27
Monoclinic, 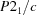

*a* = 13.8754 (15) Å
*b* = 12.5349 (13) Å
*c* = 7.7093 (8) Å
β = 93.566 (2)°
*V* = 1338.3 (2) Å^3^
*Z* = 4
Mo *K*α radiation
μ = 0.11 mm^−1^
*T* = 296 K
0.85 × 0.26 × 0.12 mm

#### Data collection

Bruker APEXII DUO CCD diffractometer
Absorption correction: multi-scan (*SADABS*; Bruker, 2009 ▶) *T*_min_ = 0.915, *T*_max_ = 0.988
17153 measured reflections
4415 independent reflections
2586 reflections with *I* > 2σ(*I*)
*R*_int_ = 0.026

#### Refinement

*R*[*F* ^2^ > 2σ(*F* ^2^)] = 0.045
*wR*(*F* ^2^) = 0.153
*S* = 1.02
4415 reflections
229 parameters
All H-atom parameters refined
Δρ_max_ = 0.21 e Å^−3^
Δρ_min_ = −0.23 e Å^−3^

### 

Data collection: *APEX2* (Bruker, 2009 ▶); cell refinement: *SAINT* (Bruker, 2009 ▶); data reduction: *SAINT*; program(s) used to solve structure: *SHELXTL* (Sheldrick, 2008 ▶); program(s) used to refine structure: *SHELXTL*; molecular graphics: *SHELXTL*; software used to prepare material for publication: *SHELXTL* and *PLATON* (Spek, 2009 ▶).

## Supplementary Material

Crystal structure: contains datablock(s) global, I. DOI: 10.1107/S1600536811039845/hb6416sup1.cif

Structure factors: contains datablock(s) I. DOI: 10.1107/S1600536811039845/hb6416Isup2.hkl

Supplementary material file. DOI: 10.1107/S1600536811039845/hb6416Isup3.cml

Additional supplementary materials:  crystallographic information; 3D view; checkCIF report

## Figures and Tables

**Table 1:** Hydrogen-bond geometry (Å, °) *Cg*1 is the centroid of the C1–C6 ring.

*D*—H⋯*A*	*D*—H	H⋯*A*	*D*⋯*A*	*D*—H⋯*A*
N2—H1*N*1⋯O1^i^	0.892 (15)	2.013 (15)	2.8841 (14)	165.2 (14)
C4—H4⋯O1^ii^	0.92 (2)	2.47 (2)	3.370 (2)	168 (2)
C1—H1⋯*Cg*1^iii^	0.98 (2)	2.92 (2)	3.7025 (18)	138.0 (15)

Symmetry codes: (i) 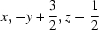; (ii) 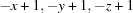; (iii) 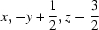.

**Fig. 1.**
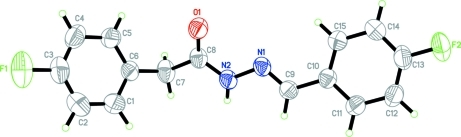
The asymmetric unit of the title compound, showing 50% probability displacement ellipsoids.

**Fig. 2.**
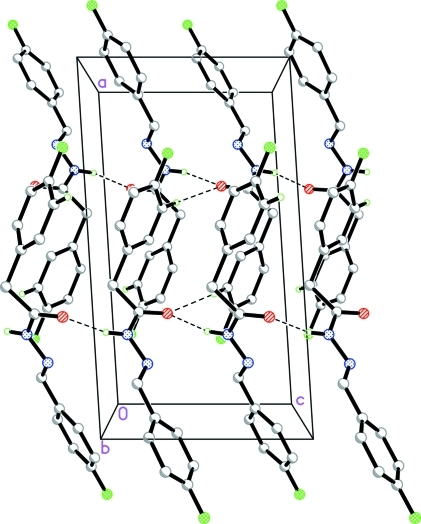
The crystal packing of the title compound (I). H atoms not involved in hydrogen bonding are omitted.

## supplementary crystallographic information

### Comment

Large number of aroylhydrozones have been synthesized in the recent years (Li & Qu, 2011; Zhang, 2011; Fan *et al.*, 2008) which can serve as intermediates in synthesizing biologically active compounds (Ajani *et al.*, 2010; Avaji *et al.*, 2009; Rasras *et al.*,  2010).

The asymmetric unit of the title compound is shown in Fig. 1. The dihedral angle between the two benzene rings (C1–C6/C10–C15) is 48.73 (8)°. The hydrazine group is twisted slightly with C9-N1-N2-C8, N1-N2-C8-C7 and N2-N1-C9-C10 torsion angles of 172.48 (12)°, 169.41 (12)° and 174.13 (11)°, respectively.

In the crystal structure, (Fig. 2),  the molecules are connected *via* intermolecular strong N—H···O and weak C—H···.O (Table 1) hydrogen bonds forming one-dimensional supramolecular chains along the *c*-axis.  The crystal structure is further stabilized by π–π interactions between the benzene (Cg2; C10–C15) rings [Cg2···Cg2 = 3.6579 (10) Å; -x, 2-y, 1-z] and C—H···π interaction involving the centroid of the C1–C6 (Cg1) ring.

### Experimental

An equimolar mixture of 2-(4-fluorophenyl)acetohydrazide and 4-fluorobenzaldehyde was refluxed for four hours in the presence of few drops of acid catalyst and ethanol as solvent.  The compound obtained was filtered, washed, dried and recrystalised from ethanol to yield colourless needles.

### Refinement

All hydrogen atoms were located from a difference Fourier maps and refined freely [N–H = 0.890 (17) Å and C–H = 0.92 (2)– 1.001 (18) Å].

### Crystal data

**Table:**

C_15_H_12_F_2_N_2_O	*F*(000) = 568
*M**_r_* = 274.27	*D*_x_ = 1.361 Mg m^−^^3^
Monoclinic, *P*2_1_/*c*	Mo *K*α radiation, λ = 0.71073 Å
Hall symbol: -P 2ybc	Cell parameters from 3867 reflections
*a* = 13.8754 (15) Å	θ = 3.1–28.1°
*b* = 12.5349 (13) Å	µ = 0.11 mm^−^^1^
*c* = 7.7093 (8) Å	*T* = 296 K
β = 93.566 (2)°	Needle, colourless
*V* = 1338.3 (2) Å^3^	0.85 × 0.26 × 0.12 mm
*Z* = 4	

### Data collection

**Table:**

Bruker APEXII DUO CCD diffractometer	4415 independent reflections
Radiation source: fine-focus sealed tube	2586 reflections with *I* > 2σ(*I*)
graphite	*R*_int_ = 0.026
φ and ω scans	θ_max_ = 31.4°, θ_min_ = 2.2°
Absorption correction: multi-scan (*SADABS*; Bruker, 2009)	*h* = −20→18
*T*_min_ = 0.915, *T*_max_ = 0.988	*k* = −18→17
17153 measured reflections	*l* = −11→11

### Refinement

**Table:**

Refinement on *F*^2^	Primary atom site location: structure-invariant direct methods
Least-squares matrix: full	Secondary atom site location: difference Fourier map
*R*[*F*^2^ > 2σ(*F*^2^)] = 0.045	Hydrogen site location: inferred from neighbouring sites
*wR*(*F*^2^) = 0.153	All H-atom parameters refined
*S* = 1.02	*w* = 1/[σ^2^(*F*_o_^2^) + (0.0752*P*)^2^ + 0.0834*P*] where *P* = (*F*_o_^2^ + 2*F*_c_^2^)/3
4415 reflections	(Δ/σ)_max_ < 0.001
229 parameters	Δρ_max_ = 0.21 e Å^−^^3^
0 restraints	Δρ_min_ = −0.23 e Å^−^^3^

### Special details

**Table:**

Geometry. All s.u.'s (except the s.u. in the dihedral angle between two l.s. planes) are estimated using the full covariance matrix. The cell s.u.'s are taken into account individually in the estimation of s.u.'s in distances, angles and torsion angles; correlations between s.u.'s in cell parameters are only used when they are defined by crystal symmetry. An approximate (isotropic) treatment of cell s.u.'s is used for estimating s.u.'s involving l.s. planes.
Refinement. Refinement of F^2^ against ALL reflections. The weighted R-factor wR and goodness of fit S are based on F^2^, conventional R-factors R are based on F, with F set to zero for negative F^2^. The threshold expression of F^2^ > 2σ(F^2^) is used only for calculating R-factors(gt) etc. and is not relevant to the choice of reflections for refinement. R-factors based on F^2^ are statistically about twice as large as those based on F, and R- factors based on ALL data will be even larger.

### Fractional atomic coordinates and isotropic or equivalent isotropic displacement parameters (Å^2^)

**Table:**

	*x*	*y*	*z*	*U*_iso_*/*U*_eq_	
F1	0.76995 (8)	0.61553 (12)	0.39014 (18)	0.1100 (4)	
F2	−0.21176 (8)	0.96296 (11)	0.46342 (17)	0.1026 (4)	
O1	0.31477 (7)	0.63023 (8)	0.33790 (11)	0.0593 (3)	
N1	0.18163 (7)	0.77410 (8)	0.22901 (12)	0.0457 (2)	
N2	0.26129 (8)	0.75056 (9)	0.13713 (13)	0.0486 (3)	
C1	0.56239 (12)	0.71146 (14)	0.1320 (2)	0.0681 (4)	
C2	0.65526 (13)	0.70642 (17)	0.2100 (3)	0.0799 (5)	
C3	0.67904 (11)	0.62143 (15)	0.3128 (2)	0.0695 (4)	
C4	0.61612 (13)	0.54169 (15)	0.3421 (2)	0.0693 (4)	
C5	0.52362 (12)	0.54705 (12)	0.2623 (2)	0.0598 (4)	
C6	0.49585 (9)	0.63228 (11)	0.15654 (15)	0.0501 (3)	
C7	0.39518 (11)	0.63844 (15)	0.07075 (17)	0.0603 (4)	
C8	0.32068 (9)	0.67230 (11)	0.19528 (14)	0.0478 (3)	
C9	0.13383 (10)	0.85672 (11)	0.17896 (16)	0.0482 (3)	
C10	0.04314 (9)	0.88361 (10)	0.25544 (15)	0.0469 (3)	
C11	0.00248 (11)	0.98366 (12)	0.22675 (18)	0.0572 (3)	
C12	−0.08399 (11)	1.01067 (14)	0.2957 (2)	0.0647 (4)	
C13	−0.12794 (11)	0.93654 (14)	0.3933 (2)	0.0655 (4)	
C14	−0.09066 (12)	0.83664 (14)	0.4254 (2)	0.0663 (4)	
C15	−0.00469 (11)	0.81012 (12)	0.35531 (19)	0.0571 (3)	
H1	0.5397 (14)	0.7715 (16)	0.059 (3)	0.094 (6)*	
H2	0.7039 (17)	0.7598 (17)	0.196 (3)	0.101 (7)*	
H4	0.6331 (15)	0.4869 (19)	0.417 (3)	0.104 (7)*	
H5	0.4770 (13)	0.4897 (15)	0.275 (2)	0.079 (5)*	
H7A	0.3934 (12)	0.6871 (14)	−0.029 (2)	0.074 (5)*	
H7B	0.3739 (14)	0.5682 (15)	0.026 (2)	0.080 (5)*	
H9	0.1566 (11)	0.9042 (12)	0.0904 (19)	0.058 (4)*	
H11	0.0356 (12)	1.0384 (14)	0.157 (2)	0.071 (5)*	
H12	−0.1126 (15)	1.0787 (17)	0.276 (2)	0.088 (6)*	
H14	−0.1233 (15)	0.7855 (16)	0.491 (3)	0.090 (6)*	
H15	0.0238 (13)	0.7400 (14)	0.374 (2)	0.073 (5)*	
H1N1	0.2685 (11)	0.7821 (12)	0.035 (2)	0.061 (4)*	

### Atomic displacement parameters (Å^2^)

**Table:**

	*U*^11^	*U*^22^	*U*^33^	*U*^12^	*U*^13^	*U*^23^
F1	0.0595 (6)	0.1415 (12)	0.1263 (9)	0.0162 (6)	−0.0142 (6)	−0.0132 (8)
F2	0.0706 (7)	0.1242 (10)	0.1173 (9)	0.0344 (6)	0.0415 (6)	0.0197 (7)
O1	0.0625 (6)	0.0731 (7)	0.0435 (5)	0.0189 (5)	0.0134 (4)	0.0069 (4)
N1	0.0455 (5)	0.0527 (6)	0.0396 (5)	0.0043 (4)	0.0094 (4)	−0.0022 (4)
N2	0.0490 (6)	0.0616 (7)	0.0362 (5)	0.0069 (5)	0.0119 (4)	0.0018 (4)
C1	0.0654 (9)	0.0731 (10)	0.0675 (9)	0.0061 (8)	0.0180 (7)	0.0172 (8)
C2	0.0626 (10)	0.0866 (13)	0.0921 (13)	−0.0099 (9)	0.0175 (9)	0.0057 (10)
C3	0.0493 (8)	0.0895 (12)	0.0701 (9)	0.0114 (8)	0.0064 (6)	−0.0068 (8)
C4	0.0693 (10)	0.0696 (10)	0.0695 (9)	0.0247 (8)	0.0079 (7)	0.0071 (8)
C5	0.0606 (8)	0.0538 (8)	0.0664 (8)	0.0082 (7)	0.0148 (7)	0.0035 (6)
C6	0.0502 (7)	0.0589 (8)	0.0427 (6)	0.0099 (6)	0.0155 (5)	−0.0009 (5)
C7	0.0545 (8)	0.0864 (11)	0.0409 (6)	0.0146 (7)	0.0107 (5)	−0.0074 (7)
C8	0.0462 (6)	0.0611 (8)	0.0367 (5)	0.0050 (6)	0.0059 (4)	−0.0054 (5)
C9	0.0515 (7)	0.0508 (7)	0.0429 (6)	0.0015 (6)	0.0071 (5)	0.0005 (5)
C10	0.0490 (6)	0.0489 (7)	0.0429 (6)	0.0039 (5)	0.0035 (5)	−0.0028 (5)
C11	0.0612 (8)	0.0547 (8)	0.0563 (7)	0.0090 (6)	0.0092 (6)	0.0071 (6)
C12	0.0660 (9)	0.0607 (9)	0.0682 (9)	0.0205 (8)	0.0103 (7)	0.0060 (7)
C13	0.0510 (8)	0.0800 (11)	0.0669 (8)	0.0149 (7)	0.0136 (6)	0.0006 (7)
C14	0.0589 (9)	0.0680 (10)	0.0736 (9)	−0.0006 (7)	0.0185 (7)	0.0084 (8)
C15	0.0564 (8)	0.0511 (8)	0.0647 (8)	0.0042 (6)	0.0115 (6)	0.0022 (6)

### Geometric parameters (Å, °)

**Table:**

F1—C3	1.3633 (18)	C6—C7	1.510 (2)
F2—C13	1.3538 (17)	C7—C8	1.5146 (17)
O1—C8	1.2268 (15)	C7—H7A	0.982 (18)
N1—C9	1.2765 (16)	C7—H7B	0.984 (19)
N1—N2	1.3812 (14)	C9—C10	1.4619 (18)
N2—C8	1.3402 (17)	C9—H9	0.973 (16)
N2—H1N1	0.890 (17)	C10—C11	1.3873 (19)
C1—C6	1.377 (2)	C10—C15	1.3947 (19)
C1—C2	1.389 (3)	C11—C12	1.384 (2)
C1—H1	0.98 (2)	C11—H11	1.001 (18)
C2—C3	1.356 (3)	C12—C13	1.363 (2)
C2—H2	0.96 (2)	C12—H12	0.95 (2)
C3—C4	1.355 (3)	C13—C14	1.372 (2)
C4—C5	1.390 (2)	C14—C15	1.380 (2)
C4—H4	0.92 (2)	C14—H14	0.95 (2)
C5—C6	1.3840 (19)	C15—H15	0.971 (18)
C5—H5	0.976 (19)		
			
C9—N1—N2	115.83 (10)	C8—C7—H7B	105.7 (11)
C8—N2—N1	118.67 (10)	H7A—C7—H7B	106.8 (15)
C8—N2—H1N1	121.3 (10)	O1—C8—N2	122.72 (11)
N1—N2—H1N1	119.7 (10)	O1—C8—C7	122.27 (12)
C6—C1—C2	121.31 (16)	N2—C8—C7	115.01 (11)
C6—C1—H1	116.0 (12)	N1—C9—C10	120.62 (12)
C2—C1—H1	122.6 (12)	N1—C9—H9	121.5 (9)
C3—C2—C1	118.30 (17)	C10—C9—H9	117.8 (9)
C3—C2—H2	117.8 (13)	C11—C10—C15	118.84 (12)
C1—C2—H2	123.9 (13)	C11—C10—C9	119.73 (12)
C4—C3—C2	122.74 (16)	C15—C10—C9	121.43 (12)
C4—C3—F1	118.37 (16)	C12—C11—C10	120.90 (14)
C2—C3—F1	118.89 (17)	C12—C11—H11	118.5 (10)
C3—C4—C5	118.51 (16)	C10—C11—H11	120.6 (10)
C3—C4—H4	120.9 (14)	C13—C12—C11	118.25 (14)
C5—C4—H4	120.5 (14)	C13—C12—H12	120.2 (12)
C6—C5—C4	120.90 (16)	C11—C12—H12	121.5 (12)
C6—C5—H5	117.8 (11)	F2—C13—C12	118.59 (15)
C4—C5—H5	121.2 (11)	F2—C13—C14	118.36 (15)
C1—C6—C5	118.24 (14)	C12—C13—C14	123.05 (14)
C1—C6—C7	120.86 (14)	C13—C14—C15	118.33 (15)
C5—C6—C7	120.90 (14)	C13—C14—H14	121.7 (12)
C6—C7—C8	112.71 (10)	C15—C14—H14	119.9 (12)
C6—C7—H7A	110.7 (10)	C14—C15—C10	120.63 (14)
C8—C7—H7A	109.8 (10)	C14—C15—H15	120.8 (10)
C6—C7—H7B	110.9 (11)	C10—C15—H15	118.5 (10)
			
C9—N1—N2—C8	172.48 (12)	C6—C7—C8—O1	−49.3 (2)
C6—C1—C2—C3	−0.4 (3)	C6—C7—C8—N2	131.47 (14)
C1—C2—C3—C4	0.0 (3)	N2—N1—C9—C10	174.13 (11)
C1—C2—C3—F1	−179.98 (16)	N1—C9—C10—C11	166.80 (12)
C2—C3—C4—C5	0.5 (3)	N1—C9—C10—C15	−14.1 (2)
F1—C3—C4—C5	−179.50 (14)	C15—C10—C11—C12	0.0 (2)
C3—C4—C5—C6	−0.6 (2)	C9—C10—C11—C12	179.11 (13)
C2—C1—C6—C5	0.3 (2)	C10—C11—C12—C13	0.3 (2)
C2—C1—C6—C7	−179.85 (14)	C11—C12—C13—F2	178.96 (15)
C4—C5—C6—C1	0.2 (2)	C11—C12—C13—C14	−0.3 (3)
C4—C5—C6—C7	−179.62 (13)	F2—C13—C14—C15	−179.36 (15)
C1—C6—C7—C8	−102.93 (16)	C12—C13—C14—C15	−0.1 (3)
C5—C6—C7—C8	76.90 (18)	C13—C14—C15—C10	0.5 (2)
N1—N2—C8—O1	−9.9 (2)	C11—C10—C15—C14	−0.4 (2)
N1—N2—C8—C7	169.41 (12)	C9—C10—C15—C14	−179.50 (14)

### Hydrogen-bond geometry (Å, °)

**Table:**

Cg1 is the centroid of the C1–C6 ring.

**Table:**

*D*—H···*A*	*D*—H	H···*A*	*D*···*A*	*D*—H···*A*
N2—H1N1···O1^i^	0.892 (15)	2.013 (15)	2.8841 (14)	165.2 (14)
C4—H4···O1^ii^	0.92 (2)	2.47 (2)	3.370 (2)	168 (2)
C1—H1···Cg1^iii^	0.98 (2)	2.92 (2)	3.7025 (18)	138.0 (15)

Symmetry codes: (i) *x*, −*y*+3/2, *z*−1/2; (ii) −*x*+1, −*y*+1, −*z*+1; (iii) *x*, −*y*+1/2, *z*−3/2.
